# Moral State of Society
‡Letters on "Labour and the Poor," published in the *Morning Chronicle* newspaper, by the Special Correspondent of that journal.


**Published:** 1850-04-01

**Authors:** 


### Art. III.—MORAL STATE OF SOCIETY.‡

The editor of the "Morning Chronicle" has done incalculable service by awakening public attention, in a series of articles on "Labour and the Poor," to the physical and moral condition of the labouring population of this country. It is impossible to estimate the benefit which will result from this *exposé*, harrowing and painful as it is to the best feelings of human nature. The consummate ability which the editor of the "Morning Chronicle" has displayed in originating in a daily paper—which, as a general rule, purposes only to give the ordinary news of the day, with comments on passing events—an investigation into the actual state of the working people of this country; and the indomitable spirit, untiring zeal, and extraordinary talent with which the gentlemen selected have carried out this most important conception, are entitled to the warmest commendation of all who wish well to the human species. There is a telling and, we may add, a touching truthfulness about the scenes described, which our best and most graphic writers of fictitious nar-

‡ Letters on "Labour and the Poor," published in the *Morning Chronicle* newspaper, by the Special Correspondent of that journal.

rative could not imitate with anything approaching to success. The immense body of valuable facts thus recorded in the columns of the *Morning Chronicle* will, we trust, form the elements or data for some most important legislative enactments, having for their object the amelioration of the sad and melancholy state of very large sections of the population of our principal agricultural and manufacturing towns. The subjoined remarks on the moral condition of society have been suggested to our mind by a perusal of the letters in question. We purpose dealing with principles: the facts are already on record.

A wretched woman died of cold and hunger in a garret, when, upon searching her corpse, there were found 300*l.* concealed among the folds of her night-cap. In yonder room is squatted an old man, with a few grey hairs straggling over his wrinkled brows, counting the pebbles given him by his keeper for the sake of peace, as he is never content unless he is telling them over and over again, and laying by twenty out of every thirty of these few worthless grains of dirt in a crack of the wainscoting, where he has a large store of them, which he gloats upon with as much despicable satisfaction as if they were so many ingots of bullion, or stock receipts for so many thousands of pounds sterling funded in the national securities. In another apartment is, sitting helpless on a chair, a man in the middle of life, paralysed in his lower limbs. He is haggard and pale—his eye glazed—his look languid. See—he is not merely paralysed in half his body, but his mind is also gone—he is a fool! That boy is the victim of an earlier vice—a maniac; and that girl, too, blanched to her very gums, is the same as he. Names might be adduced to verify more appalling cases, and many more instances might be brought forward to fill up the catalogue of similar guilt, folly, or madness; but, if the multitudes who play their parts on the active stage of the world, be wilful strangers to such facts as these, until their turn comes to exemplify the truth of the statement, it is, nevertheless, a theme only too familiar to the physician, whose benevolence towards the feelings of others will not allow him to draw aside a veil that conceals the weakness as well as the miseries, of his fellow-creatures.

The cause of this depravity is not exclusively to be sought for in the individuals themselves. We all of us live in an atmosphere, not altogether of our own creation, from which we derive our characters, habits, and thoughts, just as we draw the breath that vivifies our blood from the air about us. It begins with our birth. No sooner do we open our eyes upon the light, than we are made subject to ideas which rule our lot imperiously for the rest of our days. From hence our conduct springs, and leads to consequences involving the immediate fate or welfare of ourselves or others; and, at last, issues in a chain of events concatenating the destiny of generations for ages yet to come. Our influence survives our lives, as our minds survive our bodies. We live as much for others as for ourselves: and we are, in the main, woe-worth the day—woe-worth the deed!—the manufacturers of our own futurity. Select any one set of ideas, and work it out to its last results. Suppose a state of society, in which chastity is decried as a vice, and avarice extolled as a virtue, what will be the result? Every private family, and every public asylum, will be charged with its respective quota of avaricious maniacs and profligate fools.

There is not, and there never was, a legislator who did not labour to extirpate both the one and the other of these two tremendous vices as totally subversive of the well-being of the community he was appointed to govern. From the blasphemous saturnalia of Belshazzar, on the night when Babylon fell beneath the arms of Darius the Mede, down to the palmy days of Venice, that island-city, with her merchant-princes, and argosies, and licentious grandees, and sapient doges, and marble palaces, and solitary dungeons, *pozzi*, or wells, whose dismal apertures opened just above the level of the sullen water, nearing as it flowed beneath the Bridge of Sighs towards the green and sparkling Adriatic,—the history has been but one and the same—namely, an individualism of licentiousness and avarice, a general blaze of national splendour, followed by exhaustion and decay. Venice has passed away, and the vestiges of Babylon are scattered like broken tiles on her desert plains; but the passion that extinguished them both is alive and still upon the wing. Athens, with its Thessalian banquets and notorious courtesans (such as the *audax Pythias* of the Latin lyrist,) receiving the homage of heroes, sages, orators, and parasites, is now a dusty skeleton on her bare Acropolis; and Rome, too, with its impious repasts, and throngs of slaves, whose bodies and souls were at their master's beck and call, lies with her proud Corinthian columns prostrate along the ground; while the fiend who infested them both is upright and alert in the modern world, mocking at his crowd of dupes as he goads them to the brink of ruin, where he abandons them to their headlong fate.

It is an opinion common to mankind in all places, all ages, and all religions, that there is something in continence allied to the divinity. There is no code of legislation which has not taken pains to protect it, not as a personal virtue or accomplishment, but upon the broad ground of national prosperity and order. Among the Hebrews, a priest could not marry a repudiated woman, and the high priest could not marry a widow. The Talmud adds, that he could not possess two wives at the same time, although polygamy was winked at, if not allowed, in others ; and all who entered the sanctuary were bound to be *pure.* Among the Egyptians, the priest had only one wife. The chief priest among the Greeks was bound to observe a rigorous celibacy. Origen shows what means the pagan priest used in order to keep his vow; for antiquity everywhere acknowledged, whenever it could act in direct opposition to the loose passions of the populace, the paramount importance of chastity in the persons of its highest functionaries. The priests of Ethiopia were celibates and recluses. Virgil, in the sixth Æneid, places his chaste priests in the Elysian fields—*quique sacerdotes casti dum vita manebat.* And the priestesses at Athens, where the laws gave them the highest rank, were selected by the people, supported at the public expense, consecrated to the worship of the goddess, and bound over to absolute chastity. Pass forwards as time rolls on—look at Peru—the same idea prevails. A universal acclamation gives every honour to chastity. Although marriage is the natural condition, and a holy one too, yet on all sides virginity is valued at a higher price than the bonds of matrimony. The *professed* virgin is a superior being; let her but lose this qualification, legitimately though it be, and she degrades herself in a certain sense in the estimation of every one. In every period, there have been virgins consecrated to the divinity ; and it was a proverb, that Rome rose with Vesta, and with Vesta it fell. In the temple of Minerva, at Athens, the sacred fire was kept alight by virgins. In Peru, their misconduct was punishable with death, as at Rome. The law of Menu prescribes rules upon this article ; the voluptuous author of the Koran exalts it as the peculiar boon of obedience ; and Numa pronounced it to be holy and venerable. Tacitus mentions Occia, who presided over the College of Vestals for more than half a century, as a personage of eminent sanctity (*summa sanctimonia*), it is the language of monastic eulogy. It was a byword in Rome that the permitted marriage of vestals never turned out well. The same idea may be traced from Peru and Mexico, across the Pacific to China, and from China to India, and thence to the Cape of Good Hope.

The nations who favour population are those who have honoured this virtue the most; and it is a medical fact, almost too well known for repetition, that dissipated youths seldom become the fathers of a numerous offspring, and that a promiscuous indulgence of the passion leads to the extinction of the species. Barrenness is the blight of depravity. Terrible death-bed scenes are common among the debauched—and idiocy, or mental paralysis, is most frequent among worn-out profligates and rakes. We need not travel to oriental climes to witness scenes like these—they are at our doors; although the biting remark must needs be appended, that the oriental morals of this country are in this particular precisely those of pagan antiquity, indeed, of every country not swayed by the purely Christian idea. Open any classic author you please, and see what enormities passed current, and to what a low ebb the tide of morality had fallen among them. You cannot but blush—nay, you dare not translate aloud half-a-dozen pages from Herodotus, or some hundred lines in succession from Virgil, Juvenal, or Ovid. And yet these are the authors we place in the hands of the young, and bid them, in the words of Horace, *vos exemplaria Græca nocturna versate manu, versate diurna*—study them day and night, until, for the sake of the Latinity, they have got them by heart; as if with the purity of Latin diction, the impurity of morals was not imbibed at the same time.

In reviewing society psychologically, it is impossible not to consider it in a moral point of view. It is our duty to do so. For the regulation of the mind, which we have adopted as our peculiar office or calling in life, depends upon the moral well-being of the individual; and as the state of the various organs, more especially that of the sensorium, affects our ideas, so, in their turn, do our ideas affect the different subordinate viscera, and modify, lessen, exalt, or disturb their several functions, thereby rendering the individual more or less sound in mind or body, as the case may be. Accordingly, the importance of giving right ideas can scarcely be estimated at too high a cost—a cost, in fact, not to be appreciated until we enter the precincts of the mad-house, and there behold the terrible results of a single, solitary, exaggerated, unbridled idea. Once alter, direct, or suppress the erroneous idea, and the patient is instantly changed for the better; because, invisible as an idea may be in itself, it is in its effects tremendously agonistic on the animal economy. Speak a certain word to a child—it looks at you, the blood rushes to its face, the eyes sparkle, and the countenance glows with animation. It was only a word—a little word,—but that little word conveyed an idea which operated instantaneously and powerfully on the entire circulation of the body. You quit the child, but the idea remains, and its subsequent behaviour is the result of that single word. It is the same, on a greater scale, with masses of the population, with nations, with all the world. There is not a law, however cogent, that was ever enacted by the strongest government on earth, that could possibly withstand the slow and steady progress of a distinct idea universally prevalent. Put into array a complete army of a hundred thousand men to enforce an existing authority in opposition to a positive idea in active prevalence;—it is useless;—lances fall, artillery are powerless, the sabre is blunt, and the pointed bayonet is foiled—the serried ranks give way, and *the idea passes on.* Rome fell before the Christian idea, and modern society deliberately exposes itself to the risk of falling before the pagan idea so carefully indoctrinated in the education of its youth, whereby avarice is represented as a virtue, and chastity as a weakness or a vice. There is not at this moment a question pressing upon every government in Europe with such intense interest as that of wealth and morals—political economy and public education—individualism and communism—frugality and self-control.

It is a remark worthy of notice, that, whereas legislators and popular prejudices have, in all ages, deified chastity, so the public morals have, in the same proportion, been miserably low, if we except the Jewish and the Christian dispensations. This strange inconsistency is to be accounted for by showing that antiquity was, with the power of enacting wholesome laws, powerless in imparting an idea in exact correspondence with the laws it enacted; that the masses of the population were slaves whom it dared not franchise; and that there subsisted no teachers of philosophy, no sacerdotal sect, no public censor of morals (so much esteemed by Montesquieu in his *Esprit des Lois*), who could control the popular mind by means of a single energetic idea, except such as flattered the passions. Consequently, all classes were plunged into the most gross and infamous sensual indulgences. Tacitus, in speaking of the Germans, says, *Nemo enim illic vitia ridet, nec corrumpere et corrumpi sæculum vocatur*,—a sarcasm insinuating that if virtue was the habit of the Germans, vice was the reigning fashion at Rome. Some rich dowagers counted their years, not by the succession of consuls, but by the number of husbands whom it had been their good fortune to enjoy; and even Cicero, one of the first of statesmen, philosophers, and moralists, repudiated his wife Terentia after having been united to her for more than thirty years, and took to himself his ward, Publilia, a rich and handsome young woman, instead. The conventional manners of the ancients were unspeakably atrocious; and if we reflect on the awful amount of customary vice,—the absolute want of all consecration of purpose,—in the midst of which the great writers of antiquity flourished, the superb moral axioms that inflame the pages of a Horace, a Juvenal, a Catullus, a Cicero, a Seneca, &c., are more than enough to strike us dumb with astonishment. For it is according to our motive-ideas that we act well or ill; and so true is this, that every parent, as well as every politician, dreads the introduction of novel ideas tending to upset the established order of things, or likely to derange the social fabric of a kingdom. Thus, ancient Rome whetted the executioner's hatchet against the Christian idea throughout the empire, and England, for several centuries, felt herself under the necessity of putting in force the most stringent penal code ever framed against the idea of foreign religion. The chief art of diplomacy is the management of ideas. It was once proposed in the Roman Senate to give the slaves a particular costume, but the bill was withdrawn, simply upon the intimation that should the slaves by this means be enabled to count their numbers, they would unite, rise up in a body, and overthrow the empire. Slavery would have continued to the present hour, had not Christianity stepped forward and bestowed the manumission upon that ignominious class of men; not, indeed, by an anti-slavery agitation, but by imparting to the slaves themselves the idea of personal freedom while yet in bonds. No sooner was this idea of freedom conceived by the slave, than the shackles fell from his wrists without the effort of his striking a blow to break them asunder. Christianity, by a word, effected what Spartacus, in revolt, had failed in accomplishing against the legions of the commonwealth, commanded by Pompey. For of what use was the master's coercion over a fellow-creature, conscious of the freedom or independence of his own dignity, as a man, equally as much as his master himself? The day was won, and slavery ceased to exist of its own accord.

The passion for wealth is more generally diffused, and much less discountenanced than that of sensuality. For avarice is, as Lord Byron ironically styles it, a "*respectable, gentlemanly vice,*" while sensuality is always loathsome, except to those immediately engaged in the pursuit of its bootless chase. Consequently, the lust for gold has prevailed, unchecked, in all ages, but more especially in those preceding the epoch of the Christian revelation. At no time, however, has the rich man ever been looked upon as a contemptible member of society; for money is power; and whether we consider its display in the gardens and saloons of an Atticus, or its excessive accumulation in the coffers of a Crœsus, we cannot but conclude that such a person is in possession of a force which he may any day bring into operation, for or against us, with irresistible cogency and success. But, like all other insatiable desires that pretend to superhuman agency, a satiety of wealth is much less formidable than at the first sight it appears to be. It is often an engine too vast for the grasp of intellect, to whose management it has been entrusted—it is either hoarded or squandered; in the one case, it is calamitous; and in the other, when the necessities, together with some of the refinements of life have been supplied, nothing else remains except the vacant occupation of reckoning up the surplus of an overflowing exchequer, and striking the satisfactory balance lodged in our treasurer's hands. Life is short, and so are the means of living.* We cannot live two days in one, nor repose in two rooms at the same time; we cannot read more than one book with profit to our minds, nor eat more than one dinner a-day with benefit to our health. One hour follows another, and minute drops away after minute, just as the hour-glass distils its sand grain by grain. To possess more than is enough for the day and the evils thereof, is only an idea, and, sooth to say, it is the eccentric idea of a madman. For who knows what shall be on the morrow? and who can tell whether we shall not be numbered with the dead? or whether our money bags may not find themselves wings, and be fled? And then, in this case, the idea of our money has flown away also, and nought remains but our naked carcass, without an idea, except the idea of money, which we no longer possess. Such is the maniac whose possessions and losses have equally turned his head. Few, indeed, have merited the rare commendation of the poet—*Dîi tibi divitias dederant, artemque fruendi.*

The enormous possession of wealth never fails to lead to evils both public and private. The monarch who aggrandizes territory, or stores up money for his successor, has always bequeathed to his kingdom revolutions, wars, and corruption of morals. Henry VII. of England was a royal miser, and his son was a spendthrift and a libertine. Alexander the Great left extensive dominions, gained by conquest, which were, upon his decease, split up among several competitors, and divided to the four winds of heaven. Rich men's sons seldom prosper, and affluent families abound in a greater number

* Among some of the Dialogues of the Dead, of which Lucian's and Fénélon's are the chief, there is a story told of a ghost, who had been a long time dead, meeting a ghost recently defunct, and inquiring whether any more people were left alive in the world above? "What do you mean?" replies the second ghost. "Why," says the first, "such numbers have arrived among us lately, that there is a common report in these nether regions that the world is at last depopulated." "Depopulated!" exclaims the ghost just arrived; "I was alive yesterday, and the world never was so full; all they want is plague, pestilence, and famine, battle and murder, and sudden death, to carry off the excess of population."

of scrofulous and maniacal members than those of the poorer classes—whether we account for it by the better provision for the sickly, or the degenerating influence of a superabundance of means. The old world was swept away by a flood on account of its gross corruption of morals, and it would seem that the brute beasts that entered the ark with Noah were preferred to the preservation of the same number of human beings, cankered to the core by lust and avarice, and their constant attendant, incorrigible infidelity; for, among the great men of those days, one was a distinguished huntsman, another a metallurgist, another a musician, implying a very advanced stage of civilization and wealth. And yet, notwithstanding, within five hundred years after the deluge, two licentious cities were purposely destroyed by fire, and the first of the patriarchs, for the sake of rescuing his relative, cut off the rear-guard of the five kings banded together in a marauding party to plunder the cities of the plain, whose luxury and opulence were already sufficient to excite their cupidity. Herodotus is full of the wealth of the kings of Egypt and the monarchs of Persia. Everybody knows of Tadmor and Palmyra in the desert,—the wealth of Carthage, Tyre, and Antioch,—that of Rome during the Empire, and of Constantinople down to the close of the middle ages, when it was, after repeated attempts, at length successfully stormed and sacked by the Turks. There are the more recent examples of Spain with Mexico and Peru; Portugal with the Brazils; and maritime Genoa, whose arms shook the throne of Cantacuzene, and betrayed the debility of the eastern Cæsars. All these luxurious cities have passed away, as it were, like the dissolving-views of a dream; but the idea which animated them all still survives, and continues to do its work among the sons of men with unerring precision and effect. Along the frieze of the entablature of its temple, glitters the monosyllable *Gold,* and in the inter-columnar spaces, where the ancients imprinted the word *Salve* in mosaic, is inscribed in much more attractive characters, *The Golden Way.**

* Poor Hood has left us an incomparable lampoon on the rich fool, in his story of "Miss Kilmansegg."

"Moreover, he had a golden ass,
Sometimes at stall, and sometimes at grass,
That was worth its own weight in money—
And a golden hive on a golden bank,
Where golden bees, by alchemical prank,
Gather'd gold instead of honey.

"Gold! and gold! and gold without end!
He had gold to lay by, and gold to spend,
Gold to hive, and gold to lend,
And reversions of gold *in futuro*—

One of the immediate effects of avarice, as well as of lust, is the extinction of the sentiment of friendship. This is a very remarkable feature; and if it has been already noticed by others, it has, at least, not been prominently brought forward as the psychological symptom of a maniacal tendency. Madmen have no friends—it is utterly impossible they should have any. They congregate, indeed, together, or even mix with others of a sound mind in the world; but they cannot form a friendship, nor feel within their breasts so delightful an emotion. The reason is, that, with all their madness, they are conscious of possessing a terrible secret, which they dare not impart, or which, if imparted, would instantly excite a horrid antipathy in the person to whom it was confided. Madness is essentially selfish; and so is avarice, and so is lust; and these two passions once let loose, begin in a latent germ of madness, and, if indulged in to excess, terminate in absolute mania. For madness does, for the most part, subsist in a latent form, and so do the worst of vices. The profligate, expert at debauching innocence, can tell no one of his dastardly designs—his better friends would be obliged to shun him, while his comrades in guilt would only become competitors, or partners, in the dreadful trade. And so, likewise, the miser is a solitary man. To share his means with others is to diminish his pelf; every generous feeling goes far towards creating an extraordinary call upon his purse; and the parable of the good Samaritan must sound in his ears as a dilemma, out of which it would be his instinctive interest to extricate himself as soon as he could, at the least possible cost. Do you not see the maniacal taint in avarice and lust? Ought not St. Luke's or Bedlam to be the proper hiding-place of each?

Another symptom pathognomonic of these two mortal sins is apathy of mind and body—a condition which is likewise highly descriptive of confirmed madness. But this want of sympathy with external things comes on by degrees, and is manifested only in proportion as the nerves are exhausted by the intense operation of one long-continued idea upon the sensorium. At their accession, they are irritants, producing a paroxysm of arterial and nervous excitement, which may continue an indefinite period; but, at the last, the grey-

---

In wealth the family revell'd and roll'd,

Himself, his wife, and his son so bold;—

And his daughters sang to their harps of gold,

'O bella età dell' oro.'

"Gold! and gold! and nothing but gold!
The same auriferous shrine behold
Wherever the eye could settle!
On walls—the side board—ceiling," &c. &c.

headed miser and the jaded rake are both of them men with wasted forms and sallow visages, dry features, and lack-lustre eyes. You may see them any day in a mad-house, if not frequently enough as you pass along the crowded thoroughfares of the great city.

At their best estate, neither the one nor the other of these wretched creatures is ever exhilarated, for a time however transient, with a glow of chivalry. It is a word without a meaning in their vocabulary. The follies of knight-errantry are a fable at the present day. There is no one to throw down, none to take up, the gauntlet in defiance or defence of those virtues which own no arbiter except the point of the lance and the breast-plate of honour. It is absurd to allude to a bygone sentiment; for all is reduced to the frigid rules of good breeding, or the legal benevolence of a poor-law rate; and even the permitted vices of society are, like the stones upon the highway, *macadamized* to one and the self-same size, and carefully scattered over the surface, without leaving an individual prominence in any one of them to attract our notice or impede our way. There is a want of freedom in all they do, especially in their thoughts, fettered to one idea, and terminating at length in unequivocal monomania. Fatuity and death are the unavoidable extremity of this state of things; for recovery is hopeless, since the nervous structure has undergone such deleterious changes in the course of prolonged disease, that, to undertake to renew its vigour in the settled form of the malady, would be little else than to pretend to create the moral and intellectual faculties, together with their exquisite organizations, once more anew.

The object of the preceding imperfect observations is to point out the necessity of generating and circulating distinct ideas, especially in regard to morals, as a social and physical benefit in the education of youth. Ideology is not a novelty : it is a Platonism which captivated or incensed the sophists of Athens before Christ—agitated, six centuries later, the fickle and astute schools of the Alexandrine Platonicians ; and, in conjunction with the Aristotelian philosophy, provoked the verbal hostilities that arose to disturb the tranquillity of the learned, forced into inky array along the opposite ranks of the *Nominalists* and *Realists* of the thirteenth century. One of the immediate fruits of this subtle controversy was the *Summum Theologicum* of St. Thomas Aquinas, the angel of the schools, which, with his other works, was publicly burnt at the time of the Reformation. This composition is a masterpiece of the human understanding, expressed in language so logically exact that it defies criticism, outvies competition, and remains to this hour a pyramid of erudition pointing upwards to heaven in spirit, and resting below on its own broad and all-enduring basis of solid reasoning and incontrovertible conclusions. It solidifies thought, explodes doubt, demonstrates truth. Its tone and tendency are essentially moral or ethical; and the reader rises up from the patient perusal of it completely satisfied and permanently convinced. Well-developed ideas are sure to rivet the attention. We admire the productions of the great masters of art produced by Italy, Florence, Spain, &c., and wonder how they contrived to represent the sublime and beautiful on canvas in the consummate manner they do. Take, for instance, the Spanish painter Velasquez—look at his charming picture, *La Couronnement de la Vierge*—there are only three figures in it (*hæc decies repetita placebit*), and yet the artist was not aware of any extraordinary merit in tracing the subject, since he was only representing ideas common to himself and those for whom he painted. To these ideas we are strangers. Indeed, the abundance as well as the excellence of the ideas with which some minds are enriched is most marvellous.

Any one of the numerous airs out of *Zauberflöte* would be almost enough to establish the reputation of any ordinary composer, and yet, throughout the opera, Mozart continues to lavish a succession of melodies, replete with a profusion of the sweetest notes, amounting to prodigality. It is nearly the same with Bellini—whether we listen to *La Sonnambula,* the popular favourite—*Norma* in flats, or *Il Pirata* in sharps.

Consider, on the other hand, an instance of no ideas. A policeman was assaulted—the supposed assailants were placed at the bar of the Mansion House; and George Ruby's testimony was thought likely to cast some light on the affray. He seemed about fourteen years of age. From his answers to the questions of Alderman Humphrey, it appeared that he could not read, never said his prayers, understood nothing of the nature of prayer, knew nothing of God, and, as he expressed it, had heard of the devil, but did not know him. He sums up very briefly his small stock of information, "I knows how to sweep a crossing; that's all." He seems to have lived somewhere near Bishopsgate-street; where the "crossing" is on which all his intellect centres, we know not. Any of our readers may have given him a halfpenny, and not observed that he was more dirty, more ragged, more stamped with the marks of ignorance and degradation than half his young brethren of the broom.—(*The Times, January,* 1850.)

Consider another instance of mean ideas, arising from the worst of circumstances. There are boys who roam about the sides of the river Thames at low tide to pick up coals, bits of iron, rope, bones, and copper nails, that fall while a ship is being repaired. They are at work sometimes early in the morning, and sometimes late in the afternoon, according to the tide. They usually work from six to seven hours a day. My informant, a quick, intelligent little fellow, who has been at the business three years, tells me the reason they take to *mudlarking* is, that they are nearly all fatherless, and their mothers are too poor to keep them, so they take to it because they have nothing else to do. This boy works with about twenty or thirty mudlarks every day; and they may be seen, he tells me, at daybreak very often, with their trousers tucked up, groping about and picking out pieces of coal from the mud. They go into the river up to their knees, and in searching the mud, they very often run pieces of glass and long nails into their feet; when this is the case, they go home and dress the wounds, and return directly; for should the tide come up without their finding anything, they must starve that day. At first, it is a difficult matter to stand in the mud, and he has known many young beginners to fall in. Their average earnings are threepence a day. After they leave the river, they go home, and scrape their trousers, and make themselves as tidy as possible; they then go into the streets, and make a little by holding gentlemen's horses, or opening cab-doors. In the evening they mostly go to the ragged schools.—(*Correspondent of the Morning Chronicle.* 1849.)

But enough of scenes like these. Let us turn from the indicative past to the potential future:

> "As when a trav'ller o'er the heath'ry waste,
> Treads darkling, wearisome his nightly way,
> The dawn begins, the glorious sun ascends,
> While he, forgetful of his drear sojourn,
> Pauses and feels the magic beam of morn."

The parallel attempted to be drawn between the fall of Rome and that of some prosperous kingdoms of the present day, is a *schoolboyism* unworthy of notice. Events never repeat themselves, centuries never retrace their steps; and the overthrow of modern Europe, after the similitude of ancient Rome, is a political, a moral, a religious impossibility. It is incontestable that religion has for a long time past been gaining a decided ascendancy over men's minds and hearts—this fact is conveyed with electric despatch in every line of daily intelligence. Ordinary modern society is, with all its faults, a model of perfection, when compared with the *élite* of pagan society at its fairest epochs. The constant complaints which we are now so fond of making, respecting our defective civilization, and shameful ignorance, are a proof that these evils are rapidly advancing in the way of correction and amendment. Words mean thoughts in active operation. *Justice, humanity, morality,* the continual theme of all classes, signify that these virtues are practically on the increase throughout the world; for a principle, once proclaimed, must gradually acquire influence, and, if it be true, will, in the end, rule over all around it. We appeal to students who have had the leisure to read (*occiditque legendo*) the delightful pages of Fleury, Voltaire (*Histoire Generale*), Gibbon, Balmez, &c., whether the evidence is not conclusive in favour of the progressive amelioration of mankind. It is the duty of the patriot, says the dignified and far-sighted historian of the "Decline and Fall," to prefer and promote the exclusive interest and glory of his own country; but a philosopher may be permitted to enlarge his views, and to consider Europe as one great republic, whose various inhabitants have obtained almost the same level of politeness and cultivation. The balance of power will continue to fluctuate, and the prosperity of our own, or the neighbouring kingdoms, may be alternately exalted or depressed; but these partial events cannot essentially injure our general state of happiness, the system of arts, and laws, and manners, which so advantageously distinguish, above the rest of mankind, the Europeans and their colonies. Private genius and public industry may, for a short interval, be, in some places, suspended or extirpated; but the more necessary arts of civil life, like hardy plants, survive the tempest, and strike an everlasting root into the most unfavourable soil. The splendid days of Augustus and Trajan were eclipsed by a cloud of ignorance; and the barbarians subverted the laws and palaces of Rome. But the scythe, the invention or emblem of Saturn, still continued annually to mow the harvests of Italy; and the human feasts of the Lestrigons have never been renewed on the coasts of Campania. Since the first discovery of the arts, war, commerce, and religious zeal have diffused among the savages of the Old and New Worlds their inestimable gifts—they have been successively propagated—they can never be lost. We may therefore acquiesce in the pleasing conclusion, that every age of the world has increased, and still increases, the real wealth, the happiness, the knowledge, and perhaps the virtue, of the human race.

